# Dobutamine in the Management of Advanced Heart Failure

**DOI:** 10.3390/jcm13133782

**Published:** 2024-06-27

**Authors:** Tanjeev Ahmad, Shamitha A. Manohar, Jason D. Stencel, Thierry H. Le Jemtel

**Affiliations:** John W. Deming Department of Medicine Tulane University, 131 S., New Orleans, LA 70112, USA; smanohar@tulane.edu (S.A.M.); jstencel@tulane.edu (J.D.S.); lejemtel@tulane.edu (T.H.L.J.)

**Keywords:** decompensated heart failure with reduced ejection fraction, cardiovascular disease, advanced heart failure, inotrope, dobutamine, milrinone, levosimendan decompensated reduced ejection fraction

## Abstract

**Background**: The potential harm and clinical benefits of inotropic therapy in patients with decompensated heart failure with reduced ejection fraction or advanced heart failure were debated for three decades. Nonetheless, confronted with a dismal quality of life in the last months to years of life, continuous home inotropic therapy has recently gained traction for palliative therapy in patients who are not candidates for left ventricular mechanical circulatory support or heart transplantation. **Methods**: As continuous inotropic therapy is only considered for patients who experience symptomatic relief and display objective evidence of improvement, clinical equipoise is no longer present, and randomized controlled trials are hard to conduct. **Results**: We first outline the transient use of inotropic therapy in patients with decompensated heart failure with reduced ejection fraction and emphasize the hemodynamic requisite for inotropic therapy, which is a demonstration of a low cardiac output through a low mixed venous oxygen saturation. Lastly, we review the current experience with the use of home inotropic therapy in patients who are not candidates or are awaiting mechanical circulatory support or heart transplantation. **Conclusions**: Evidence-based clinical data are needed to guide inotropic therapy for refractory decompensated heart failure with reduced ejection fraction in patients who are ineligible or awaiting mechanical circulatory support or heart transplantation.

## 1. Intravenous Inotropic Therapy

Positive inotropic therapy has been and remains the subject of controversy in the treatment of heart failure (HF) [1]. Thirty years ago, the detrimental effects of oral milrinone on mortality and morbidity in the Prospective Randomized Milrinone Evaluation Survival Evaluation (PROMISE) trial and intravenous (IV) milrinone in the Outcomes of a Prospective Trial of Intravenous Milrinone for Exacerbations of Chronic Heart Failure (OPTIME-CHF) study tempered the initial enthusiasm for positive inotropic therapy in the management of decompensated heart failure with reduced ejection fraction (HFrEF) [2,3]. Subsequently, three post hoc analyses of landmark randomized controlled trials (RTCs) questioned the safety of positive inotropic therapy in decompensated HFrEF [4,5,6].

In the Flolan International Randomized Survival Trial (FIRST), decompensated HFrEF patients who were receiving dobutamine had a worse outcome than that of patients who did not after adjustment for age, sex baseline functional status/left ventricular ejection fraction, randomization to epoprostenol, and ischemic cause of HF [4]. However, no specific adjustment addressed the decision to add dobutamine to the management of decompensated HFrEF before the randomization to epoprostenol or to standard care. The decision was likely related to a markedly depressed cardiac output (CO).

Next, in the Randomized Evaluation of Mechanical Assistance in treatment of Chronic heart Failure (REMATCH) trial, where 71% of patients receiving IV inotropic therapy at randomization and 29% of patients were not receiving IV inotropic therapy, two or more inotropic agents were administered at randomization in 43% of patients; 75% of patients received dobutamine, 43% received milrinone, and 30% received dopamine [5]. Patients not receiving IV inotropic therapy at randomization had higher survival rates independent of Left Ventricular Assist Device (LVAD) implantation. As in the post hoc analysis of the FIRST trial, no specific adjustment addressed the rationale for inotropic therapy before the randomization to LVAD or to continued medical therapy.

Although positive inotropic therapy may hasten the progression of the underlying myocardial disease in patients with HF, it alleviates symptoms and returns decompensated HFrEF to a compensated state when optimal neuro-hormonal modulation fails or cannot be attempted due to systemic hypotension [7]. Nowadays, intravenous (IV) positive inotropic therapy is commonly considered in three clinical situations. The first is the escalation to inotropic therapy in patients hospitalized for decompensated HFrEF who do not respond to or do not tolerate optimal doses of guideline-directed medical therapy (GDMT). The second situation is palliative inotropic therapy in hospitalized patients for decompensated HFrEF who, not candidates for LVAD or heart transplantation (HT), experience marked symptomatic deterioration after any attempt to taper or discontinue IV inotropic therapy [8,9]. The third situation is patients approved for heart transplantation who are awaiting a donor heart.

## 2. Aims and Safety of Intravenous Inotropic Therapy

The cardinal hemodynamic aim of positive inotropic therapy is to restore a normal or near-normal CO. A sine qua non requisite of positive inotropic therapy is the demonstration of a low CO [10]. Regrettably, and likely due to spiraling costs, Medicare no longer requires demonstration of hemodynamic changes when initiating home inotropic therapy [11].

The measurement of CO by thermodilution requires the insertion of a pulmonary artery catheter, which, although routinely performed in the intensive care unit, is expensive. Alternatively, the insertion of a peripherally inserted central catheter (PICC line) is a less expensive and safer procedure than pulmonary artery catheterization, which provides an estimate of CO through measurement of central vein oxygen saturation (ScvO_2_%).

Half a century ago, ScvO_2_ was found to reflect myocardial function in patients admitted to an intensive care unit for acute myocardial infarction [12]. Vein O_2_ saturation was >60% in AMI patients without evidence of myocardial dysfunction, ≤60% in AMI patients with clinical evidence of HF, and ≤45% in AMI patients in shock, as evidenced by a blood pressure ≤90 mmHg and decreased peripheral perfusion. Subsequently, several studies underlined the poor correlation between ScvO_2_ and mixed venous O_2_ saturation (SvO_2_) after cardiac surgery or in critically ill patients [13,14,15,16,17]. Nonetheless, many still view ScvO_2_ as a useful estimate of Svo_2_, although ScvO_2_ and SvO_2_ may differ [18,19,20,21].

In healthy individuals, O_2_ saturation is greater in the inferior vena cava (IVC) than the superior vena cava (SVC) due to the contribution of highly O_2_ saturated blood from the renal veins to the IVC [22]. In patients with HF or shock, O_2_ saturation is lower in IVC than in SVC due to a marked reduction in renal perfusion and preferential cerebral perfusion [23]. Hence, O_2_ saturation in the SVC (ScvO_2_) is usually 5% higher than in the IVC in patients with HF or shock. Further, contrary to SvO_2_, ScvO_2_ does not reckon the contribution of low O_2_ saturated (40–50%) blood from the coronary sinus [14,24]. Central vein O_2_ saturation (ScvO_2_) can underestimate SvO_2_ by 10–20% in patients with cardiogenic shock [13]. The right atrium (RA) drains blood from the SVC, IVC, and coronary sinus. Hence, advancing the central vein catheter into the right atrium (RA) obliterates the difference between ScvO_2_ and SvO_2_ [13,21,25]. Last, femoral vein O_2_ saturation poorly correlates with ScvO_2_ in critically ill patients [26]. Sepsis, a left-to-right shunt, or a systemic inflammatory syndrome should be excluded in patients with decompensated HFrEF and ScvO_2_ or SvO_2_ >60% [27,28]. When ScvO_2_ is <50%, patients who are hospitalized for decompensated HFrEF should undergo right heart catheterization to confirm or exclude a low CO state. In summary, while ScvO_2_ is an imperfect surrogate for SvO_2_, it should be obtained when the insertion of a pulmonary artery catheter is not feasible.

The limited energy supplies of a depressed myocardium hinder the use of IV-positive inotropes, especially dobutamine, in patients with advanced HF. By increasing myocardial contractility and heart rate, IV-positive inotropes tend to increase myocardial oxygen consumption (MVO_2_), as the left ventricular (LV) wall stress may not decrease sufficiently to offset the metabolic cost of increasing myocardial contractility and heart rate [29]. The metabolic cost of IV dobutamine is clearly of concern in patients with ischemic cardiomyopathy. It also concerns patients with dilated cardiomyopathy, as blood flow in the LV subendocardial layer is as reduced in dilated cardiomyopathy as in ischemic cardiomyopathy [30]. In a pig model of LV systolic dysfunction, MVO_2_ increases out of proportion to hemodynamic changes when dobutamine is administered at high doses [31]. At low doses, dobutamine does not waste oxygen in the same model [31]. At a rate of infusion of 10 mcg/kg/min, dobutamine increases MVO_2_ by 54% [32]. At a rate of infusion of 5.0 mcg/kg/min, dobutamine does not alter heart rate or blood pressure, while it decreases pulmonary capillary wedge pressure and increases CO [33]. Coronary blood flow increases proportionally to the rise in CO, and the coronary arterial–venous difference does not change, resulting in a 20% increase in MVO_2_ [33]. Hence, dobutamine needs to be infused at the lowest rate of infusion that provides the needed CO improvement. We seldom use dobutamine at rates of infusion >5.0 mcg/kg/min to prevent large increases in MVO_2_, ventricular arrhythmias, and hemodynamic tolerance [34,35].

Last, positive inotropic agents like dobutamine and milrinone worsen the defective intracellular [Ca^2+^] handling of patients with HF. [33] By increasing intracellular diastolic [Ca^2+^] positive inotropic agents, especially dobutamine, this increases the risk of fatal ventricular arrhythmias at higher rates of infusion. The addition of oral amiodarone to long-term intermittent dobutamine infusion lowers the risk of death from any cause (hazard ratio, 0.403; 95% confidence interval [CI] (0.164–0.048) over a follow-up of 2 years in a randomized controlled double-blind trial of 30 patients with advanced HF. The median survival time was 574 days with the addition of oral amiodarone and 144 days with placebo [36]. Besides oral amiodarone and close monitoring of the serum potassium concentration, the only effective intervention for the treatment of dobutamine-induced ventricular tachycardia/fibrillation is an implantable cardioverter defibrillator (ICD).

## 3. Escalation to Dobutamine in Hospitalized Patients with Advanced Heart Failure

The choice of a positive inotropic agent between dobutamine, milrinone, and levosimendan depends on clinical situations and physician preferences. When long-term beta-adrenergic blockade (BAB) cannot be tapered and discontinued due to coexisting conditions like atrial fibrillation (AF) or portal hypertension, milrinone and levosimendan are the preferred inotropic agents. Further, investigators prefer milrinone to dobutamine in patients with biventricular failure and pulmonary hypertension. Milrinone exerts a positive inotropic effect at low doses (intravenous [IV] boluses of 25.0 mcg/kg) [37]. At higher doses (IV boluses of 75 mcg/kg), milrinone exerts potent vasodilator effects [37]. Milrinone increases myocardial contractility at low doses that do not affect heart rate or blood pressure. The positive inotropic effect of milrinone is modest on the right ventricle (RV) [38]. In a subset of patients with isolated RV failure, milrinone was not superior to dobutamine in the Dobutamine Compared with Milrinone (DOREMI) trial [39]. The DOREMI trial compared milrinone to dobutamine in patients with cardiogenic shock (CS) stages B, C, D, or E with a combined endpoint of in-hospital death, cardiac arrest, heart transplantation, need for circulatory support, transient ischemic attack/stroke, or renal replacement therapy [34]. Aside from the infrequent use of pulmonary artery catheters, the limited use of mechanical circulatory support in stages B and C of CS (12% in patients randomized to milrinone and 15% in patients randomized to dobutamine) does not reflect contemporary practice in the United States [40,41]. Dobutamine had no significant advantage over milrinone or vice versa in the DOREMI trial [39]. However, due to the dismal prognosis of patients in CS, the DOREMI trial was not powered to detect a small treatment effect [39]. A meta-analysis of nine studies and two randomized controlled trials of milrinone versus dobutamine in patients in low CO or in CS revealed that dobutamine may be associated with a shorter length of hospitalization and a greater all-cause mortality [42].

Levosimendan exerts a positive inotropic action through calcium sensitization of troponin C, a vasodilator action through the activation of ATP-sensitive potassium channels in smooth vascular muscle cells, and a protective action through the activation of ATP-sensitive mitochondrial potassium channels (Table 1) [43].

From 2007 to 2021, six major randomized controlled trials of levosimendan were reported [44,45,46,47,48,49]. Three trials were positive [44,45,46]. Compared to dobutamine, levosimendan reduced mortality in advanced HF patients receiving beta-adrenergic blockade, while intermittent use of levosimendan reduced functional decline and hospitalizations for HF, along with the incidence of acute HF decompensation [44,45,46]. Three trials were neutral or negative [47,48,49]. Levosimendan did not reduce all-cause mortality compared to dobutamine, while intermittent use of levosimendan did not improve a composite endpoint of death/LVAD/HT nor improved functional capacity and quality of life compared to a placebo. In contrast to randomized controlled trials, systematic reviews, meta-analyses, and single-center experiences uniformly reported that the intermittent use of levosimendan is safe and improves morbidity and mortality in patients with advanced HF, providing great attention is given to patient dosing, dosing intervals, and patient monitoring [50,51,52,53,54]. When intravenously administered at 0.2 mcg/kg/min for a total dose of 6.25 mg every 2 weeks, levosimendan facilitates medical optimization in advanced HF patients who are intolerant to GDMT [55]. Last, compared to dobutamine, the use of levosimendan appears to have increased from 2012 to 2021 in patients with cardiogenic shock [56]. Levosimendan is marketed worldwide but is not currently approved in the United States by the Food and Drug Administration (FDA). A randomized, placebo-controlled trial of oral levosimendan is currently underway in the United States for the treatment of pulmonary hypertension in patients with HF with preserved ejection fraction. Currently, dobutamine and milrinone are the only two available positive inotropic agents for the treatment of decompensated HFrEF, or long-term inotropic support, in the United States.

A short half-life is the overwhelming advantage of dobutamine over other inotropic agents. The short half-life of dobutamine allows rapid titration to an effective and safe rate of infusion as well as the elimination of dobutamine within 5–10 min of an adverse effect. Dobutamine-mediated symptomatic relief needs to be corroborated by consonant hemodynamic changes. The positive inotropic action of dobutamine aims to increase CO through an increase in stroke volume rather than heart rate. Dobutamine commonly increases the heart rate in patients with advanced HF and atrial fibrillation. In patients with ischemic cardiomyopathy, a 10–15% increase in heart rate may require reducing the rate of infusion of dobutamine, while a similar increase in heart rate may be tolerated in patients with dilated cardiomyopathy. Dobutamine at a rate of infusion of 10 mcg/kg/min recently demonstrated a large increase in cardiac output in young patients after heart transplantation [57].

**Figure 1:**
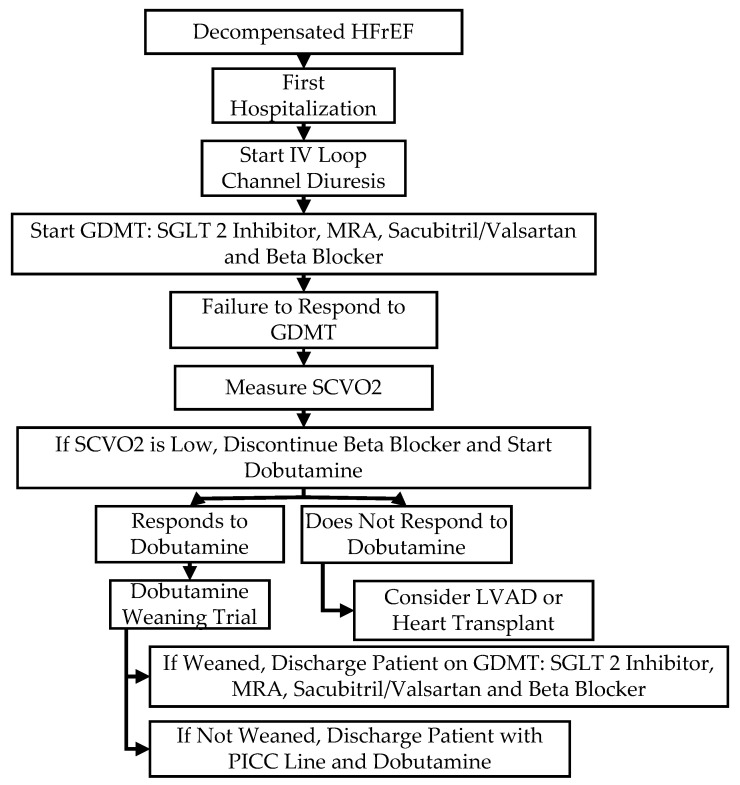
Flowchart of sequence of events for initial hospitalization of advanced HF.

**Figure 2:**
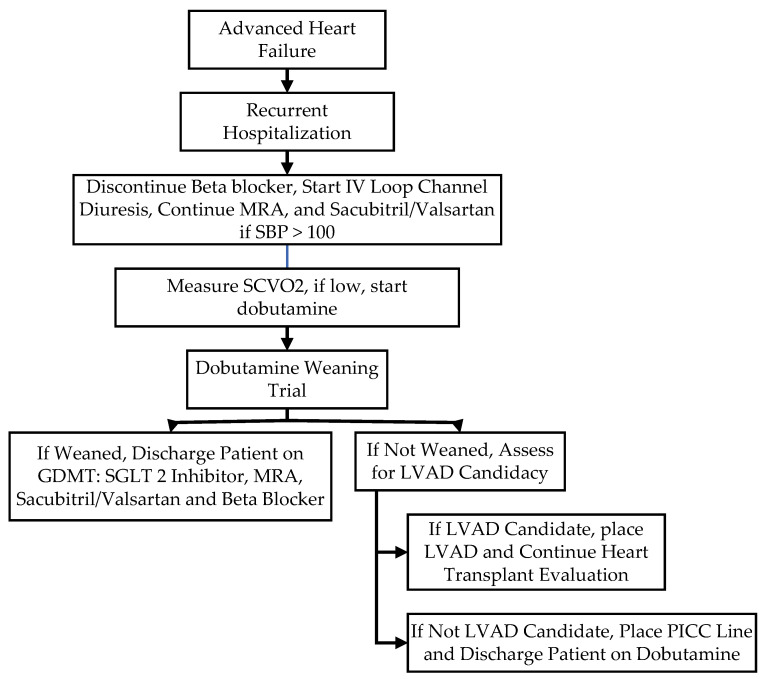
Flowchart of sequence of events for recurrent hospitalization of advanced HF.

The initiation and continuation of dobutamine therapy vary based on clinical scenarios. In patients with a first hospitalization for decompensated HFrEF and renal insufficiency, dobutamine therapy may not be considered for several days as satisfactory decongestion necessitates prolonged bed rest and close titration of the loop diuretic regimen (Figure 1). In contrast to a first hospitalization for advanced HF decompensation, dobutamine therapy may be initiated early after admission in patients with recurrent hospitalizations for advanced HF and continued until full decongestion and a return to baseline renal function are achieved (Figure 2). Slow tapering of dobutamine therapy at a rate of 1 mcg/kg/min per 6, 12, or 24 h may result in a shorter hospitalization than accelerated dobutamine discontinuation [36]. Last, BAB may not be resumed for 1–2 weeks until complete recovery and regain of baseline status.

## 4. Palliative Dobutamine Therapy

Despite the above-mentioned safety concerns, home IV inotropic therapy has increasingly been considered an integral part of palliation over the past 15 years [58,59]. Of the 98 patients who received IV-positive inotropes as part of palliative management from January 2007 to March 2013 at the University of Alabama in Birmingham, 84.8% and 15.2% received milrinone and dobutamine, respectively [37]. Notably, 80.7% of patients who received positive inotropes remained on BAB. The possibility to continue BAB underlies the preferential use of milrinone over dobutamine. Unfortunately, continuing BAB thwarts any further analysis, as patients who tolerate BAB are likely to be less sick than patients who cannot no longer tolerate BAB.

Continuous IV administration of milrinone or dobutamine had similar effects on clinical outcome in 121 patients hospitalized with advanced HF at the Cleveland Clinic [38]. However, prior to the initiation of continuous IV inotrope therapy, 5% of patients who received dobutamine were kept on BAB, and 34% of patients who received milrinone were kept on BAB. Hence, patients who received milrinone or dobutamine had a similar clinical outcome, although patients who received dobutamine were likely to be sicker than those who received milrinone. Among the 49 patients who received palliative IV-positive inotropic therapy from January 2011 to January 2017 at Columbia University Irving Medical Center, 6.1% received dobutamine and 77.6% received milrinone [39]. Besides the preference to continue BAB in the framework of palliative therapy, the preferential use of milrinone over dobutamine is unexpected for financial reasons [40]. Medicare spent an average of USD 64,152 per patient for home milrinone therapy, compared to USD 848 for home dobutamine therapy [9].

Two recent single-center observational studies investigated the safety and effectiveness of dobutamine for palliative therapy in patients with advanced HF. In the East Limburg Hospital, continuous IV administration of dobutamine alleviated symptoms and reduced HF hospitalizations at 3, 6, and 12 months, as well as health-care-related costs, in 21 consecutive patients with advanced HF [60]. The rate of dobutamine infusion was 4.0 mcg/kg/min. The mortality rate was 48% at 1 year, with 75% of patients dying at home. Nineteen patients with advanced HF were discharged home from the Hospices Civils de Lyon while receiving dobutamine at a mean rate of infusion of 2.6 mcg/kg/min. The median follow-up was 203 days (79–434). At 3, 6, and 12 months, 74, 53, and 32% of patients were alive, respectively.

In a systematic literature search of 10 studies of patients with acutely decompensated HF and CS, including 1 randomized controlled trial, milrinone lowered the risk of in-hospital mortality compared to dobutamine in all patients with acutely decompensated HF with a relative risk [RR] of 0.86, 95% CI: 0.79–0.95; *p* < 0.05) [61]. The benefit of milrinone over dobutamine was marginal in patients with acutely decompensated HF with destination therapy (RR of 0.76 95%, CI: 0.60–0.96, *p* < 0.05). In-hospital mortality was similar with milrinone and dobutamine in patients with acutely decompensated HF and CS, as well as in patients with acutely decompensated HF awaiting HT [61].

Retrospective analyses report higher survival rates with milrinone than with dobutamine in patients who are hospitalized for advanced HF and are discharged home in the framework of palliative therapy [42,43]. However, all these retrospective analyses have the same shortcomings as the post hoc analyses of the FIRST, REMATCH, and ESCAPE landmark RCTs. The clinical and laboratory parameters that led to the selection of milrinone or dobutamine cannot be retrospectively addressed.

In brief, in the absence of evidence-based data, the experience of the practitioner remains a critical resource. The successful management of positive inotropic therapy for patients with advanced HF hinges on continuity of care and close patient monitoring. Despite the challenges of conducting randomized controlled trials in sick and unstable patients, further evidence-based data are unquestionably needed. Due to a lack of standardization surrounding the measurement of patient-centered outcomes in studies of inotropes for advanced HF, the effect of inotropes on patient-reported health status remains unknown [62].

## Figures and Tables

**Table 1:** Mechanism of action of levosimendan, dobutamine, and milrinone.

	Phosphodiesterase III Inhibitor	β1 and β2 Agonist	Troponin C Synthesizer	ATP-Sensitive K Channel
Levosimendan	x		x	x
Dobutamine		x		
Milrinone	x			

## Data Availability

The original contributions presented in the study are included in the article, further inquiries can be directed to the corresponding author.
